# Glial cell derived neurotrophic factor prevents western diet and palmitate-induced hepatocyte oxidative damage and death through SIRT3

**DOI:** 10.1038/s41598-022-20101-1

**Published:** 2022-09-23

**Authors:** Simon Musyoka Mwangi, Ge Li, Arun Balasubramaniam, Didier Merlin, Paul A. Dawson, Young C. Jang, C. Michael Hart, Mark J. Czaja, Shanthi Srinivasan

**Affiliations:** 1 Division of Digestive Diseases, Department of Medicine, Emory University School of Medicine, 615 Michael St, Suite 201, Atlanta, GA 30322 USA; 2 Atlanta VA Health Care System, Decatur, GA USA; 3 Department of Medicine, Division of Pulmonary, Allergy, Critical Care and Sleep Medicine, Emory University, Atlanta, GA USA; 4 Department of Pediatrics, Division of Gastroenterology, Hepatology, and Nutrition, Emory University, Atlanta, GA USA; 5 Institute for Biomedical Sciences, Center for Inflammation, Immunity and Infection, Digestive Disease Research Group, Georgia State University, Atlanta, GA USA; 6 School of Biological Sciences and Parker H. Petit Institute for Bioengineering and Bioscience, Georgia Institute of Technology, Atlanta, GA USA

**Keywords:** Non-alcoholic fatty liver disease, Metabolic syndrome

## Abstract

Nonalcoholic fatty liver disease (NAFLD) is associated with increased oxidative stress that leads to hepatocyte and mitochondrial damage. In this study we investigated the mechanisms involved in the induction of oxidative stress and impairment of mitochondrial quality control and mitophagy in hepatocytes by the saturated fatty acid palmitate and Western diet feeding in mice and if their harmful effects could be reversed by the neurotrophic factor glial cell derived neurotrophic factor (GDNF). Western diet (WD)-feeding increased hepatic lipid peroxidation in control mice and, in vitro palmitate induced oxidative stress and impaired the mitophagic clearance of damaged mitochondria in hepatocytes. This was accompanied by reductions in hepatocyte sirtuin 3 (SIRT3) deacetylase activity, gene expression and protein levels as well as in superoxide dismutase enzyme activity. These reductions were reversed in the liver of Western diet fed GDNF transgenic mice and in hepatocytes exposed to palmitate in the presence of GDNF. We demonstrate an important role for Western diet and palmitate in inducing oxidative stress and impairing mitophagy in hepatocytes and an ability of GDNF to prevent this. These findings suggest that GDNF or its agonists may be a potential therapy for the prevention or treatment of NAFLD.

## Introduction

Nonalcoholic fatty liver disease and high-fat diet (HFD)-induced obesity in rodents are associated with mitochondrial dysfunction^1–3^. This has been suggested to be due to increased hepatic oxidative stress resulting in part from defective mitochondrial respiratory chain in the face of increased β-oxidation, and from increased peroxisomal fatty acid oxidation^2,4^. A key feature of the mitochondrial dysfunction is reduction in the activity of the mitochondria matrix-based NAD-dependent deacetylase sirtuin 3 (SIRT3)^5–8^. This is accompanied by hyperacetylation and inhibition of the activity of several mitochondrial proteins including enzymes involved in the removal of reactive oxygen species (ROS) such as the superoxide dismutases (SODs) and increased hepatic lipid peroxidation and DNA damage^2,3,7–11^. The overexpression of SIRT3, on the other hand, lowers ROS production and restores antioxidant activity in hepatocytes following exposure to the saturated fatty acid palmitic acid (PA)^11^.

Mitochondria constantly undergo a quality control process through fusion, fission and mitophagy (selective removal of mitochondria through autophagy) to maintain a healthy population of mitochondria^12^. In NAFLD and HFD models of obesity, dysregulated mitochondrial fission, fusion, and mitophagy have been suggested to contribute to the mitochondrial dysfunction observed in these conditions^13–15^. Thus, mechanisms to reduce oxidative stress and to improve mitochondria quality control could have therapeutic value in treating and preventing NAFLD.

We have previously shown that the transgenic overexpression in mice of GDNF, a neurotrophic factor that is also involved in enteric neuronal development, is protective against HFD-induced hepatic steatosis and that hepatocytes from HFD-fed GDNF transgenic (GDNF Tg) mice have higher mitochondrial β-oxidation rates than HFD-fed control (CNTRL) mice^16–18^. We have also shown that GDNF enhances autophagic flux in hepatocytes in response to exposure to PA and is protective against PA-induced lipotoxicity^18^. Whether GDNF protection against lipotoxicity and improvement of mitochondrial function involves the enhanced removal of toxic quantities of ROS and damaged mitochondria through mitophagy is not known. In this study we investigated whether GDNF can enhance the removal of ROS, and whether GDNF increases mitophagy in hepatocytes to eliminate damaged mitochondria. We show that GDNF is protective against Western diet induced hepatic oxidative damage and PA-induced increase in ROS and impairment of mitophagic flux. We also show that GDNF is protective against PA-induced reduction in hepatocyte SOD enzyme activity and SIRT3 deacetylase activity. We show that knockout of SIRT3 expression abolishes GDNF protection against palmitate-induced hepatocyte death. Finally, we show that GDNF enhances hepatocyte levels of key proteins involved in mitochondrial quality control. Our findings demonstrate an important ability of GDNF to regulate hepatocytes’ mitochondrial function to protect against palmitate-induced oxidative stress and impairment of mitophagy.

## Results

### Western diet and palmitate-induced oxidative damage and impairment of mitophagy in hepatocytes is reversed by GDNF

**Figure 1:**
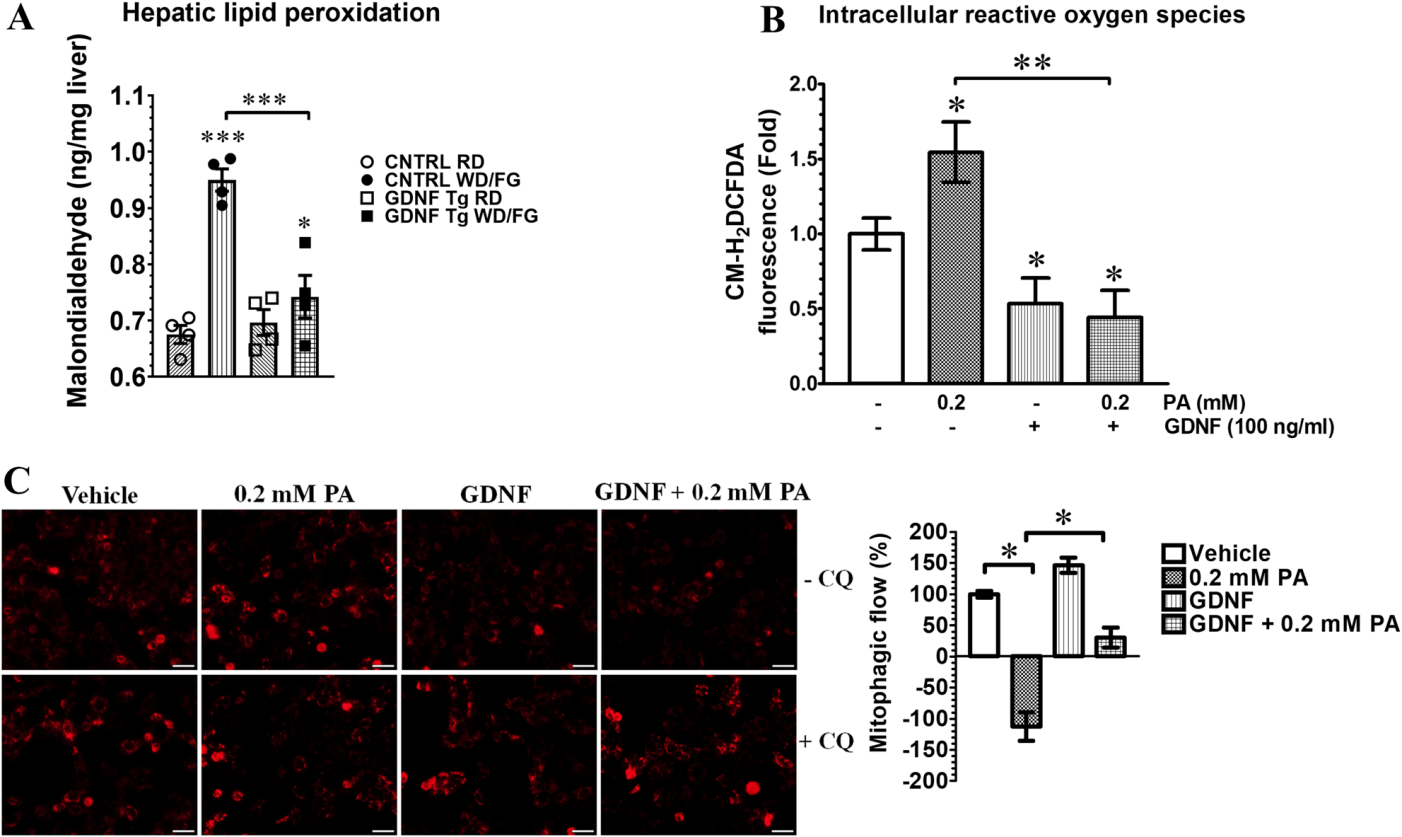
GDNF reverses Western diet- and palmitate-induced oxidative damage and impairment of mitophagy in hepatocytes. (**A**) Hepatic malondialdehyde (MDA) levels in control (CNTRL) and GDNF transgenic (GDNF Tg) mice fed regular diet (RD) or Western diet with fructose and glucose in drinking water (WD/FG) for 25 weeks. Plotted are means + SE. ***P < 0.001; *P < 0.05, relative to RD-fed control mice. N = 4 mice per group. (**B**) Intracellular ROS levels measured as CM-H_2_DCFDA fluorescence intensity in rat hepatocytes cultured for 24 h in the presence and absence of palmitate and GDNF. Plotted are means ± SE. **P < 0.01, *P < 0.05 relative to Vehicle-treated hepatocytes; N = 5. (**C**). Rat hepatocytes cultured for 6 h in the presence or absence of palmitate (PA), GDNF, and the lysosomal inhibitor chloroquine (CQ) and stained with MitoTracker Red CMX-ROS and plot of mitophagic flow expressed as % of Vehicle. Scale, 50 µm. Plotted are means ± SE. *P < 0.05; N = 2 repeats.

High levels of saturated fatty acids such as those in Western diets and high fat diets induce oxidative stress and damage in hepatocytes due to the intracellular buildup of ROS resulting from increased mitochondrial fatty acid β-oxidation^3,9,19,20^. We investigated the ability of Western diet feeding and PA to induce oxidative damage and to impair mitophagy in hepatocytes and if GDNF can protect against these harmful effects. Control mice fed a Western diet (WD) and provided with drinking water supplemented with fructose and glucose (FG) for 25 weeks had significantly (P < 0.001) higher hepatic levels of malondialdehyde (MDA) (an indicator of lipid peroxidation) indicative of increased hepatic oxidative damage when compared to control mice fed a regular rodent diet (RD) (Fig. 1A). In comparison, WD/FG-fed GDNF Tg mice had MDA levels that were significantly lower than those of WD/FG-fed control mice and only slightly higher than those of RD-fed control and GDNF Tg mice (Fig. 1A). We exposed RALA255-10G rat hepatocytes in vitro to PA and GDNF and assessed changes in intracellular ROS levels as well as the rate of mitophagic clearance of damaged mitochondria to determine if GDNF could prevent the accumulation of toxic levels of ROS and impairment of mitophagy resulting from the increased β-oxidation. We observed significant (P < 0.05) increase in ROS levels in hepatocytes exposed for 8 h to 0.2 mM PA and significant decrease in hepatocytes exposed to 0.2 mM PA in the presence of GDNF as well as in hepatocytes exposed to GDNF alone (Fig. 1B). The rate of mitophagic clearance was also significantly lower in hepatocytes exposed for 6 h to 0.2 mM PA when compared to hepatocytes exposed to Vehicle (Fig. 1C) indicative of impaired mitophagy. This decrease was, however, prevented in hepatocytes exposed to 0.2 mM PA in the presence of GDNF (Fig. 1C).

### GDNF increases hepatocyte SOD activity and protects against PA and Western diet-induced loss of SIRT3

**Figure 2:**
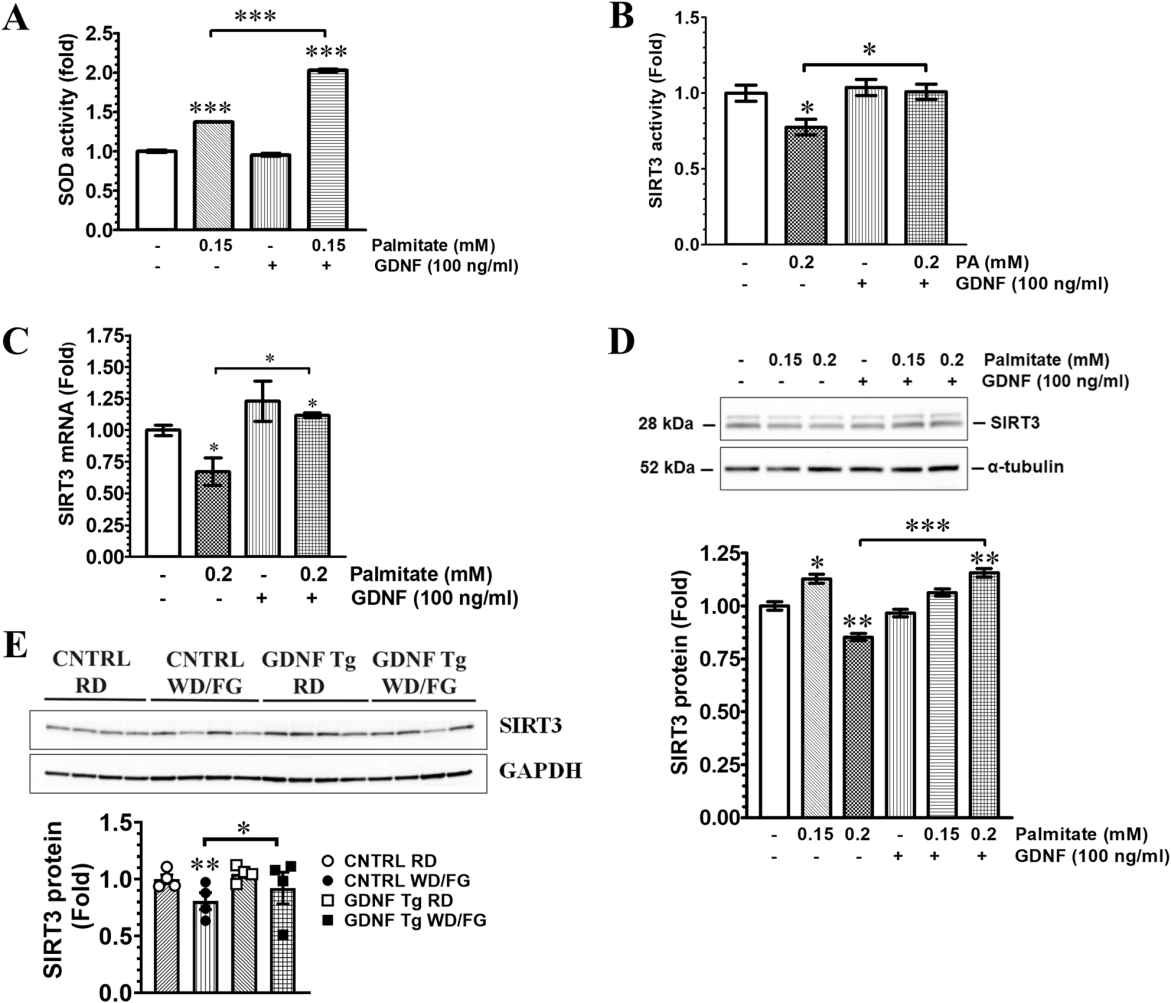
GDNF increases hepatocyte SOD activity and protects against PA and Western diet-induced loss of SIRT3. (**A**) Total SOD activity levels in rat hepatocytes cultured for 24 h in the presence or absence of palmitate (PA) and GDNF. Plotted are means ± SE. ***P < 0.001, relative to vehicle; N = 3. (**B**) SIRT3 deacetylase activity levels in rat hepatocytes cultured for 24 h in the presence or absence of palmitate and GDNF. Plotted are means + SE. *P < 0.05, relative to Vehicle-treated hepatocytes; N = 3. Analysis of SIRT3 mRNA (**C**) and protein (**D**) levels in primary human hepatocytes cultured for 24 h in the presence and absence of palmitate (PA) and GDNF. The bands were cropped from the original blots presented in Supplementary Fig. S1. Protein loading was adjusted by analyzing for α-tubulin levels. Plotted are means + SE. ***P < 0.001; **P < 0.01; *P < 0.05, relative to Vehicle-treated hepatocytes; N = 4. (**E**) Western blot analysis of hepatic SIRT3 and GAPDH (loading control) protein levels in control (CNTRL) and GDNF transgenic (GDNF Tg) mice fed regular diet (RD) or Western diet with fructose and glucose in drinking water (WD/FG) for 25 weeks. The bands were cropped from the original blots presented in Supplementary Fig. S2. Plotted are means + SE. ***P < 0.001; *P < 0.05, relative to RD-fed control mice. N = 4 mice per group. NB.

The first critical step in the detoxification of reactive superoxide radicals released during β-oxidation is catalyzed by two SOD enzymes, SOD1 which is localized mostly in the cytoplasm, but also in the mitochondrial intermembrane space, and SOD2 (manganese superoxide dismutase) which is localized in the mitochondrial matrix and mitochondrial inner membrane^21–25^. We assessed hepatocyte SOD activity to determine if GDNF’s ability to lower intracellular ROS levels was associated with increased SOD activity. Total SOD activity increased 40% in rat hepatocytes cultured for 24 h in medium supplemented with 0.15 mM PA and 100% in hepatocytes cultured in medium supplemented with 0.15 mM PA in the presence of GDNF (Fig. 2A) indicating an ability of GDNF to further enhance cellular antioxidant capacity to counter the oxidative stress induced by PA. The activity of the SOD enzymes is regulated through deacetylation of key lysine residues which is mediated by the NAD-dependent deacetylase SIRT3^26^. We investigated the effects of GDNF on SIRT3 protein levels and deacetylase activity to determine if there was a correlation between the observed GDNF-induced increase in SOD activity and SIRT3 levels. SIRT3 activity levels were significantly (P < 0.05) decreased in rat hepatocytes exposed for 24 h to 0.2 mM PA while no changes were observed in hepatocytes exposed to PA in the presence of GDNF (Fig. 2B). We also observed significant reductions in SIRT3 gene expression and modest reductions in protein levels in primary human hepatocytes cultured in medium supplemented with 0.2 mM PA alone but significant increases in both gene expression and protein levels in hepatocytes cultured in medium with 0.2 mM PA in the presence of GDNF (Fig. 2C,D). We also assessed the effects of WD/FG on hepatic SIRT3 levels. Hepatic SIRT3 protein levels in WD/FG-fed control mice were significantly (P < 0.001) reduced when compared to RD-fed control mice in agreement with previously published data^7,27^. SIRT3 levels in the liver of WD/FG and RD-fed GDNF Tg mice were, however, unchanged relative to control mice (Fig. 2E).

### GDNF reverses Western diet-induced suppression of hepatic PTEN-induced putative kinase 1 levels

**Figure 3:**
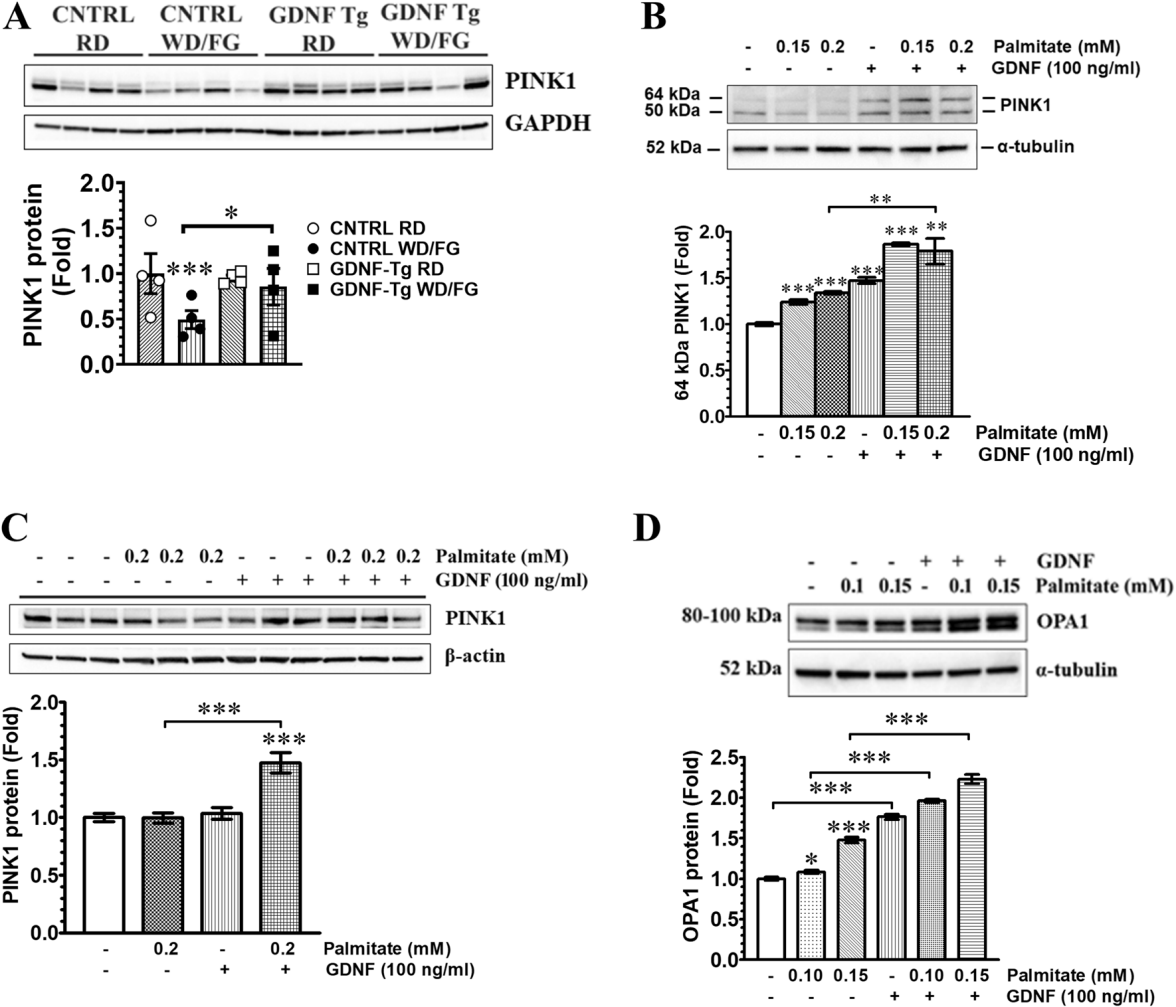
GDNF reverses Western diet-induced suppression of hepatic PTEN-induced putative kinase 1 (PINK1) levels. (**A**) Western blot analysis of hepatic PINK1 and GAPDH (loading control) protein levels in control (CNTRL) and GDNF transgenic (GDNF Tg) mice fed regular diet (RD) or Western diet with fructose and glucose in drinking water (WD/FG) for 25 weeks. The bands were cropped from the original blots presented in Supplementary Fig. S3. Plotted are means + SE. ***P < 0.001, relative to RD-fed control mice. N = 4 mice per group. (**B**). Western blot analysis of PINK1 and α-tubulin (loading control) protein levels in human hepatocytes exposed for 24 h to PA in the presence or absence of GDNF. The bands were cropped from the original blots presented in Supplementary Fig. S4. Plotted are means ± SE. ***P < 0.001; **P < 0.01, relative to vehicle and 0.2 mM PA + GDNF, respectively; N = 3. Western blot analysis of (**C**) PINK1 and β-actin (loading control) and (**D**) OPA1 and α-tubulin (loading control) protein levels in rat hepatocytes exposed to PA and GDNF for 24 h. The bands were cropped from the original blots presented in Supplementary Figs. S5 and S6. Plotted are means ± SE. ***P < 0.001, *P < 0.05, relative to Vehicle; N = 4.

We also investigated the effects of Western diet and PA on some key proteins involved in mitochondrial quality control. PTEN-induced putative kinase 1 (PINK1) is a mitochondrial protein that accumulates on the outer mitochondrial membrane marking them for removal through mitophagy. The liver of WD/FG-fed control mice had significantly (P < 0.001) reduced PINK1 (64 kDa) levels relative to RD-fed control mice while the liver of WD/FG-fed GDNF Tg mice showed no significant changes (Fig. 3A). In human and rat hepatocytes exposed for 24 h to 0.15 mM and 0.2 mM PA, the levels of the 64 kDa PINK1 protein were unchanged and significantly increased in hepatocytes exposed to palmitate in the presence of GDNF (Fig. 3B,C). The levels of OPA1 mitochondrial dynamin like GTPase, another mitochondrial membrane protein that is involved in mitochondrial fusion under certain stress conditions, were increased in rat hepatocytes after 24 h exposure to PA and increased further in hepatocytes exposed to PA in the presence of GDNF (Fig. 3D). These results demonstrate an important role for GDNF in regulating the levels of proteins involved in sensing mitochondrial stress and damage.

### Knockdown of SIRT3 expression abolishes GDNF’s protection against palmitate-induced hepatocyte cell death

**Figure 4:**
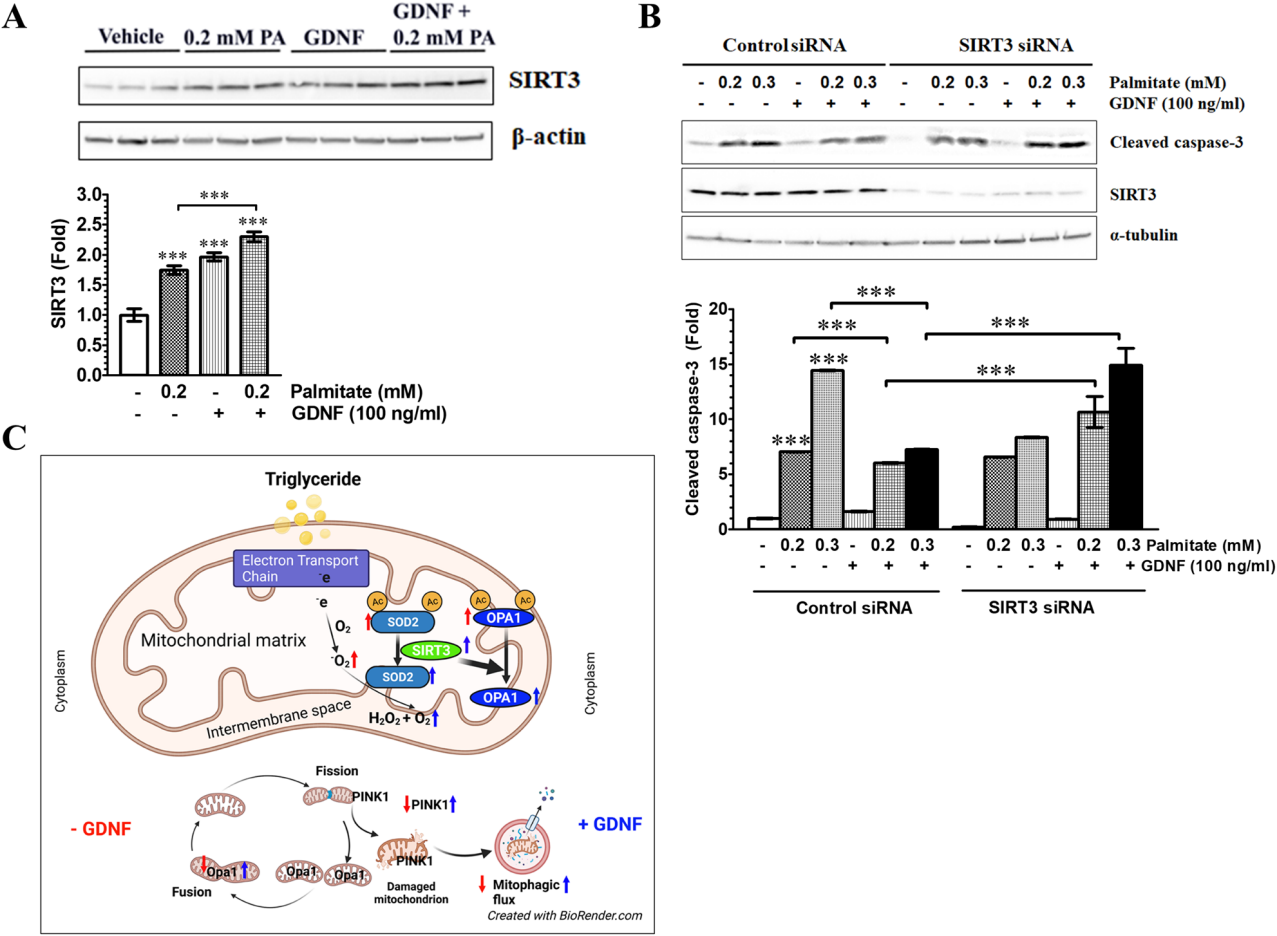
Knocked down of SIRT3 expression abolishes GDNF protection against palmitate-induced hepatocyte cell death. (**A**) Western blot analysis of SIRT3 and β-actin (loading control) protein levels in rat hepatocytes exposed to PA and GDNF for 24 h. The bands were cropped from the original blots presented in Supplementary Fig. S7. Plotted are means ± SE. ***P < 0.001, relative to Vehicle; N = 4. (**B**) Western blot analysis of SIRT3, cleaved caspase-3 and α-tubulin (loading control) levels in control siRNA and SIRT3 siRNA-transfected rat hepatocytes after 24 h exposure to palmitate (PA) and GDNF. The bands were cropped from the original blots presented in Supplementary Fig. S8. Plotted are means + SE. ***P < 0.001, relative to Vehicle-treated hepatocytes; N = 3. (**C**) Summary of proposed mechanism of GDNF action to protect hepatocytes against oxidative stress resulting from increased β-oxidation involving enhancement of superoxide dismutase (SOD) activity and SIRT3 levels as well as enhancement of resistance against fatty acid-induced suppression of mitophagy. To counter the increased mitochondrial generation of reactive oxygen species (ROS) GDNF increase SIRT3 levels and deacetylase activity which results in enhanced activity of mitochondrial SOD enzymes and their catalysis of the breakdown of ROS. GDNF also enhances mitochondrial cycling and removal of excess and damaged mitochondria through mitophagy.

Since we observed a strong ability of GDNF to enhance SIRT3 protein levels and deacetylase activity, using the RALA255-10G rat hepatocyte cell line we investigated if this conferred any survival advantage. We observed significant (P < 0.001) increases in SIRT3 protein levels in non-transfected hepatocytes cultured for 24 h in medium supplemented with 0.2 mM PA and further increases in hepatocytes cultured in medium supplemented with PA in the presence of GDNF or GDNF alone (Fig. 4A). This observation, although opposite to the previous observations in primary human hepatocytes, is not unique as increased hepatic SIRT3 gene expression and protein levels have been reported following 1 week of HFD feeding^7^. We then examined if reducing hepatocyte SIRT3 levels using siRNA can impair the ability of GDNF to protect against PA-induced cell death. GDNF was protective against PA-induced cell death in rat hepatocytes transfected with non-coding control siRNA as evidenced by the lower cleaved caspase-3 levels relative to hepatocytes cultured in the presence of PA alone (Fig. 4B). This protection was, however, lost in hepatocytes transfected with SIRT3 siRNA (Fig. 4B) thus demonstrating the important role of SIRT3 in mediating GDNF’s protection against PA-induced hepatocyte cell death. The proposed mechanism how GDNF acts to reduce oxidative stress and enhance mitophagic flux and survival in hepatocytes is summarized in Fig. 4C.

## Discussion

In this study we explored the role of a Western diet and the saturated fatty acid palmitate in inducting oxidative damage in hepatocytes and the ability of the neurotrophic factor GDNF to enhance mitochondrial quality control and prevent oxidative damage.

In NAFLD, hepatocytes respond to increased cellular free fatty acid (FFA) levels arising from FFA uptake, adipose tissue lipolysis and de novo lipogenesis by increasing the rate of mitochondrial β-oxidation which in turn results in increased intracellular ROS levels^28,29^. Increased oxidative stress with decreased SIRT3 protein and activity levels have been reported during NAFLD and in HFD rodent models of obesity^2,3,7,9,11,26,30^. This has been attributed in part to a defect in the mitochondrial respiratory chain resulting uncontrolled intracellular ROS levels^4^. The importance of SIRT3 in protecting hepatocytes against oxidative stress has been demonstrated in studies in which knockout of SIRT3 has been shown to result in the hyperacetylation of mitochondrial proteins including manganese superoxide dismutase and complex I of the electron transport chain^6,7,26,31^ while the transgenic overexpression of SIRT3 prevents oxidative stress by increasing antioxidant enzyme levels and reducing ROS levels^11^. SIRT3 acts to reduce ROS levels by deacetylating and activating SOD2^26^.

Mitophagy has been demonstrated to play an important role in protecting against the initial insult resulting from oxidative stress and changes in the levels of proteins involved in sensing mitochondrial damage such as PINK1 and in driving mitochondrial fusion and fission have been studied in HFD-fed mice. In HFD-fed mice, changes in hepatic PINK1 and OPA1 levels involving slight increases in PINK1 levels and decreases in OPA1 levels have been reported^13^. In addition, increases in the hepatic levels of other proteins involved in mitochondrial fission and fusion including dynamin related protein 1 and mitofusin 1 and reductions in the levels of protein involved in mitophagy have also been reported in HFD-fed mice^15^.

In this study we observed an important role of GDNF in preventing hepatic lipid peroxidation and oxidative stress in hepatocytes by reducing reactive oxygen species levels and enhancing mitophagic flux and the expression of proteins involved in mitochondrial quality control. These effects of GDNF occurred in the background of enhanced SOD and SIRT3 activity suggesting that GDNF possibly acts through SIRT3 to protect against oxidative stress. Indeed, when we knocked out SIRT3 expression we observed an inability of GDNF to protect against PA-induced lipotoxicity. In our study hepatic PINK1 levels were highly elevated in RD and WD/FG-fed GDNF transgenic mice. In addition, PINK1 and OPA1 protein levels were modestly increased, respectively, in human and rat hepatocytes exposed to palmitate alone and highly elevated in hepatocytes exposed to PA in the presence of GDNF. Moreover, hepatocytes exposed to GDNF alone also had highly elevated PINK1 and OPA1 levels.

Our study, thus, demonstrates an important role of GDNF in regulating hepatocyte ROS and SIRT3 levels, mitochondrial cycling and mitophagy and in protecting against WD- and palmitate-induced oxidative stress and cell death. Hence, GDNF agonists may be potential therapy for the prevention or treatment of NAFLD.

## Materials and methods

### Animals

This study followed ARRIVE guidelines on the use of experimental animals. Animal studies were conducted in 5–6 weeks old female CF-1 (control) mice and GDNF transgenic (GDNF Tg) littermates. GDNF transgenic mice are on a CF-1 background and overexpress GDNF in cells expressing glial fibrillary acidic protein (GFAP) which is expressed in several tissues including the liver^16,18^. The mice were maintained on a 12 h light–dark cycle in a temperature-controlled barrier facility with free access to food and water. Four control (CF-1) mice and 4 GDNF Tg mice were assigned to a regular rodent diet (RD) (2018SX; Teklad Global 18% Protein Extruded Rodent Diet, Harlan Laboratories, Madison, WI; 6.2% fat by weight/18% kcal from fat) with regular drinking water and maintained on the diet for 25 weeks. Another 4 CF-1 mice and 4 GDNF Tg mice were assigned to a Western Diet (WD) (TD.120528, Harlan; 21.2% fat by weight/42% kcal from fat, with increased Sucrose, and 1.25% cholesterol) along with drinking water supplemented with a high sugar solution (FG) [23.1 g/L d-fructose (Sigma-Aldrich, cat. #1286504 USP) and 18.9 g/L d-glucose (Sigma-Aldrich, cat. #1181302 USP)]^32^. These mice were also maintained on the diet for 25 weeks. The complete composition of the diets is shown in Table 1. All animal studies were approved by the Atlanta Veteran Affairs Medical Center Institutional Animal Care and Use Committee and conducted according to the recommended guidelines.

**Table 1:** Rodent diet composition.

Diet	Kcal from protein (%)	Kcal from carbohydrates (%)	Kcal from fat (%)	Fat % by weight	Total saturated fatty acids (%)	Total mono-unsaturated fatty acids (%)	Total poly-unsaturated fatty acids (%)	Cholesterol (g/kg)	Sucrose (g/kg)
2018SX (Regular diet)	24	58	18	6	0.9	3.4	1.3	0	
TD.120528 (Western diet)	15.2	42.7	42.0	21.2	61.8	27.3	4.7	12.5	405.36

### Cell culture

RALA255-10G rat hepatocytes were cultured in Gibco Dulbecco’s modified Eagle’s medium (DMEM) high glucose, HEPES, without pyruvate (#12430054, Life Technologies Corp, Grand Island, NY, USA) as previously described^33^. Human hepatocytes (H1000.H15B+, Lot No. HC3-37, Sekisui XenoTech, Kansas City, KS, USA) were cultured according to the vendors instructions. Cell culture media were replaced with fresh media every 48 h.

The rat and human hepatocytes were each assigned to 4 treatment groups: Vehicle, Palmitate, GDNF, and PA plus GDNF. Stock (6 mM) palmitate (Sigma-Aldrich, St. Louis, MO, USA) conjugated to fatty acid-free bovine serum albumin (BSA) (Sigma-Aldrich) was prepared as previously described^34^ and used at final concentration of 0.1–0.3 mM. Recombinant human and mouse GDNF (Shenandoah Biotechnology, Warwick, PA, USA) were used at a final concentration of 100 ng/mL.

### Assessment of oxidative stress

Intracellular reactive oxygen species (ROS) levels were assessed in RALA255-10G rat hepatocytes cultured for 8 h in medium supplemented with or without palmitate (PA) and GDNF. The hepatocytes were stained with the general reactive oxygen species probe CM-H_2_DCFDA (Molecular Probes, Eugene, OR, USA) according to the manufacturer’s instructions and fluorescence measured on a SpectraMax iD3 Multi-Mode Microplate Reader (Molecular Devices, San Jose, CA, USA). Lipid peroxidation was assessed in mice liver tissues using a Lipid Peroxidation (MDA) Assay Kit (#MAK085, Sigma-Aldrich).

### Assessment of antioxidant and SIRT3 activity levels

Total cellular superoxide dismutase activity was assessed in RALA255-10G rat hepatocytes cultured for 24 h in medium supplemented with or without GDNF and palmitate using the Superoxide Dismutase (SOD) Colorimetric Activity Kit (# EIASODC, Life Technologies Corp. Frederick, MD, USA). SIRT3 deacetylase activity was also assessed in RALA255-10G rat hepatocytes cultured for 24 h in medium supplemented with or without GDNF and palmitate using a fluorometric SIRT3 Activity Assay Kit (# ab156067, Abcam, Waltham, MA, USA).

### Assessment of mitophagic flux

Mitophagic flow was assessed by flow cytometry using a modification of a previously published protocol^35^. RALA255-10G rat hepatocytes were cultured for 6 h with or without palmitate (0.2 mM), GDNF (100 ng/mL), and with or without the lysosomal inhibitor chloroquine (CQ) (30 µM) (Cell Signaling Technology, Danvers, MA, USA). MitoTracker Red CMX-ROS (#M7512, Molecular Probes, Eugene, OR, USA) was then added to the cells at a final concentration of 25 nM in culture medium and the cells cultured for a further 15 min. The cells were washed once in PBS, stained for 30 min with LIVE/DEAD Fixable Near-IR Stain (#L34976, Life Technologies Corp., Carlsbad, CA, USA) and fixed with 3.7% formaldehyde in complete culture medium at 37 °C for 15 min. After washing 3 times with PBS with 1% bovine serum albumin, the cells were analyzed by flow cytometry at the Emory Pediatric and Winship Flow Cytometry Core (Emory University, Atlanta, GA, USA). Mitophagic flow was calculated from the difference in the number of MitoTracker Red CMX-ROS-positive hepatocytes between cells cultured in the presence of the inhibitor (CQ) and those cultured without CQ for each treatment.

### Western blotting

Western blotting was performed as previously described^17^ using rabbit primary antibodies to SIRT3 (#5490, Cell Signaling Technology, Danvers, MA, USA), OPA1 (#80471, Cell Signaling Technology), cleaved caspase-3 (Asp175) (Cell Signaling Technology) and PINK1 (Sigma-Aldrich) diluted 1:1000. Mouse monoclonal primary antibodies to α-tubulin (DM1A) (Cell Signaling Technology) and β-actin (A5441, clone AC-15) (Sigma-Aldrich) were diluted, respectively, 1:1000 and 1:5000 before use. Horseradish peroxidase conjugated anti-mouse and anti-rabbit IgG (Cell Signaling Technology) secondary antibodies were used at 1:2,000 dilution. A semi quantitative measurement of band intensity was performed using the Carestream Molecular Imaging Software (Carestream Molecular Imaging, New Haven, CT, USA) and Fiji^36^.

### Gene expression analysis

Total RNA was isolated using the RNeasy Mini kits (Qiagen GmbH, Hilden, Germany) and first-strand cDNA synthesized using SuperScript VILO (Invitrogen Life Technologies, Grand Island, NY, USA). Real-time PCR reactions were set up using Fast SYBR Green Master Mix (Applied Biosystems, Foster City, CA, USA) human SIRT3 upstream (5′-ATCGATGGGCTTGAGAGAGTGTC-3′) and downstream (5′-AACCCTGTCTGCCATCACGT-3′) primers and human GAPDH upstream (5′-AGCCTCAAGATCATCAGCAATGCC-3′) and downstream (5′-TGTGGTCATGAGTCCTTCCACGA-3′) primers. The primers were designed such that at least one primer in the pair spanned an intron to prevent it from priming on to genomic DNA. The inability of these primers to amplify genomic DNA was confirmed by PCR. Thermal cycling was performed on a StepOnePlus Real-Time PCR System (Applied Biosystems).

### siRNA transfection

Rat hepatocytes were transfected with SMARTpool, ON-TARGETplus Rat Sirt3 siRNA (Catalog # L-084761-03-0005; Dharmacon, Cambridge, United Kingdom) or ON-TARGETplus Non-targeting Control Pool (Catalog # D-001810-10-05) using Lipofectamine RNAiMax (Invitrogen Life Technologies) according to recommended procedure.

### Statistical analysis

Statistical analyses were conducted using the GraphPad Prism software version 3.00 for Windows (GraphPad Software, San Diego, CA). Data were tested for normality and subjected to unpaired *t* test or one-way ANOVA with Tukey posttest.

## Supplementary Information

Supplementary Information.

## Data Availability

All data generated or analyzed during this study are included in this published article (and its Supplementary Information file).

## Abbreviations

SIRT3: Sirtuin 3
PINK1: PTEN-induced putative kinase 1
GDNF: Glial cell derived neurotrophic factor
NAFLD: Nonalcoholic fatty liver disease
ROS: Reactive oxygen species
PA: Palmitate
OPA1: OPA1 mitochondrial dynamin like GTPase
HFD: High-fat diet
CM-H_2_DCFDA: 6-Chloromethyl-2′,7′-dichlorodihydrofluorescein diacetate, acetyl ester
CNTRL: CF-1 control mice
EM: Emission
EX: Extinction
FA: Fatty acid
FG: Fructose/glucose
GDNF Tg: GDNF transgenic mice
RD: Regular diet
TG: Triglyceride
WD: Western diet
