# Supplementary material for: Glial cell derived neurotrophic factor prevents western diet and palmitate-induced hepatocyte oxidative damage and death through SIRT3
Source: Sci Rep. 2022 Sep 23;12:15838. doi: 10.1038/s41598-022-20101-1 (PMC9508117; doi:10.1038/s41598-022-20101-1)
Supplement: Supplementary file 1 — Supplementary Information. [file 41598_2022_20101_MOESM1_ESM.pdf]

Supplementary Information

Glial Cell Derived Neurotrophic Factor Prevents Western diet and Palmitate-Induced Hepatocyte Oxidative damage and death through SIRT3

Simon Musyoka Mwangi[1,2]; Ge Li[1,2]; Arun Balasubramaniam[1,2]; Didier Merlin[2,5]; Paul A. Dawson[4]; Young C. Jang[6]; C. Michael Hart[2,3]; Mark J. Czaja[1]; Shanthi Srinivasan[1,2*].

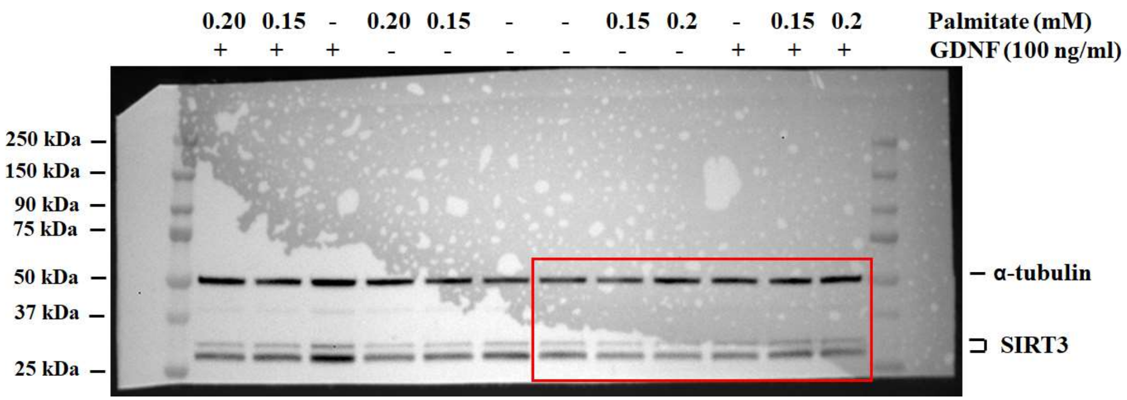

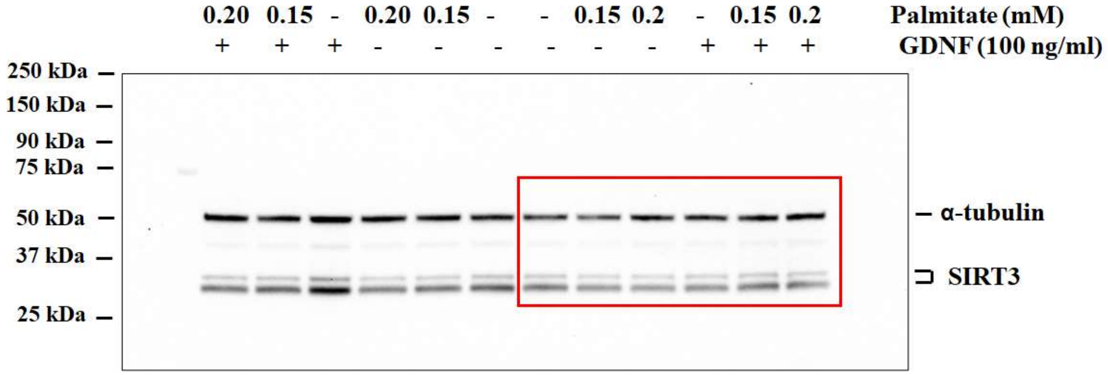

S1. Western blot analysis of SIRT3 and α-tubulin (loading control) protein levels in primary human hepatocytes cultured for 24h in the presence and absence of palmitate (PA) and GDNF. Boxed area is where bands in Fig 2D were cropped from.

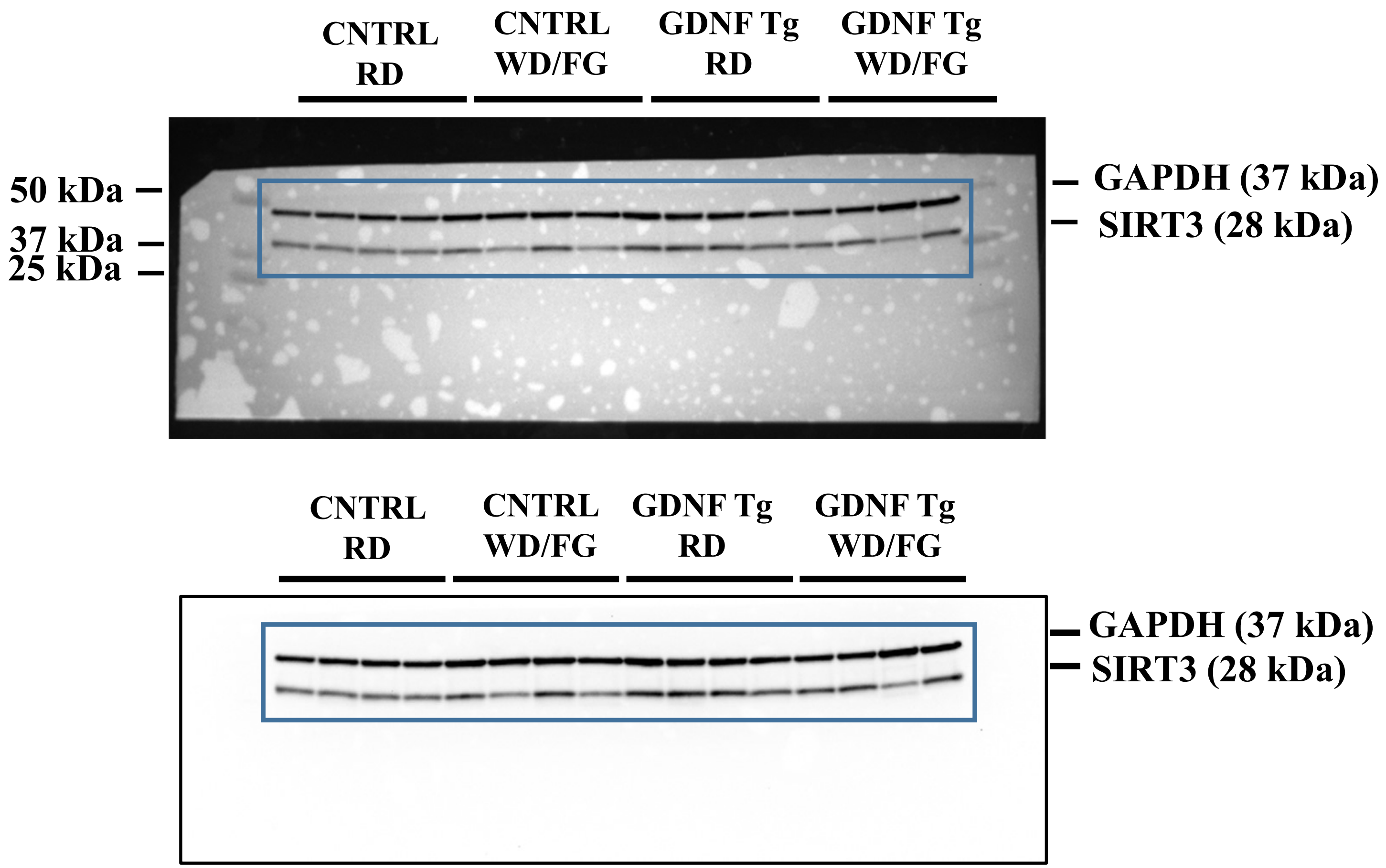

S2. Western blot analysis of hepatic SIRT3 and GAPDH (loading control) protein levels in control (CNTRL) and GDNF transgenic (GDNF Tg) mice fed regular diet (RD) or a Western diet with fructose and glucose in drinking water (WD/FG) for 25 weeks. Boxed area is where bands in Fig 2E were cropped from.

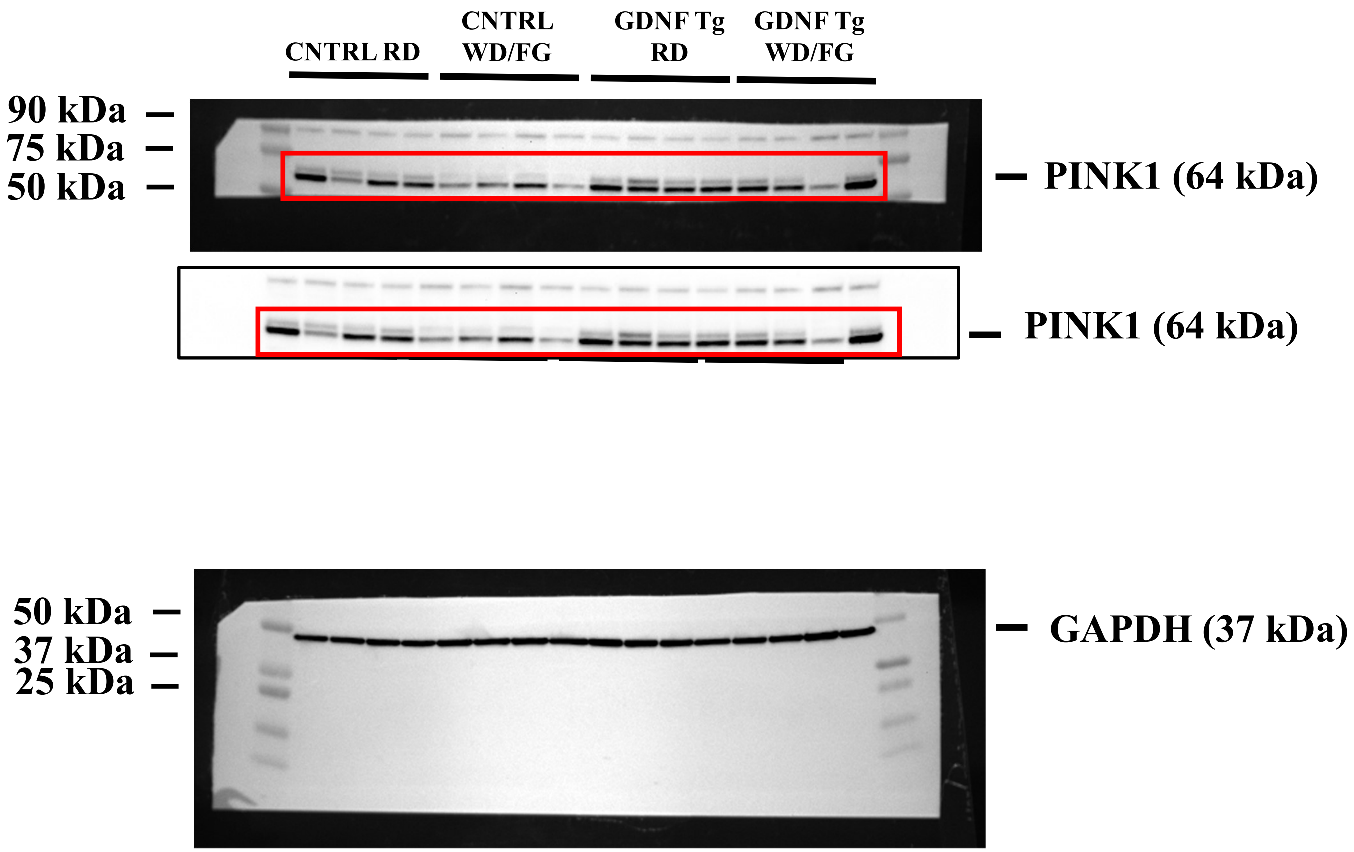

S3. Western blot analysis of hepatic PINK1 and GAPDH (loading control) protein levels in control (CNTRL) and GDNF transgenic (GDNF Tg) mice fed regular diet (RD) or a Western diet with fructose and glucose in drinking water (WD/FG) for 25 weeks. Boxed area is where bands in Fig 3A were cropped from.

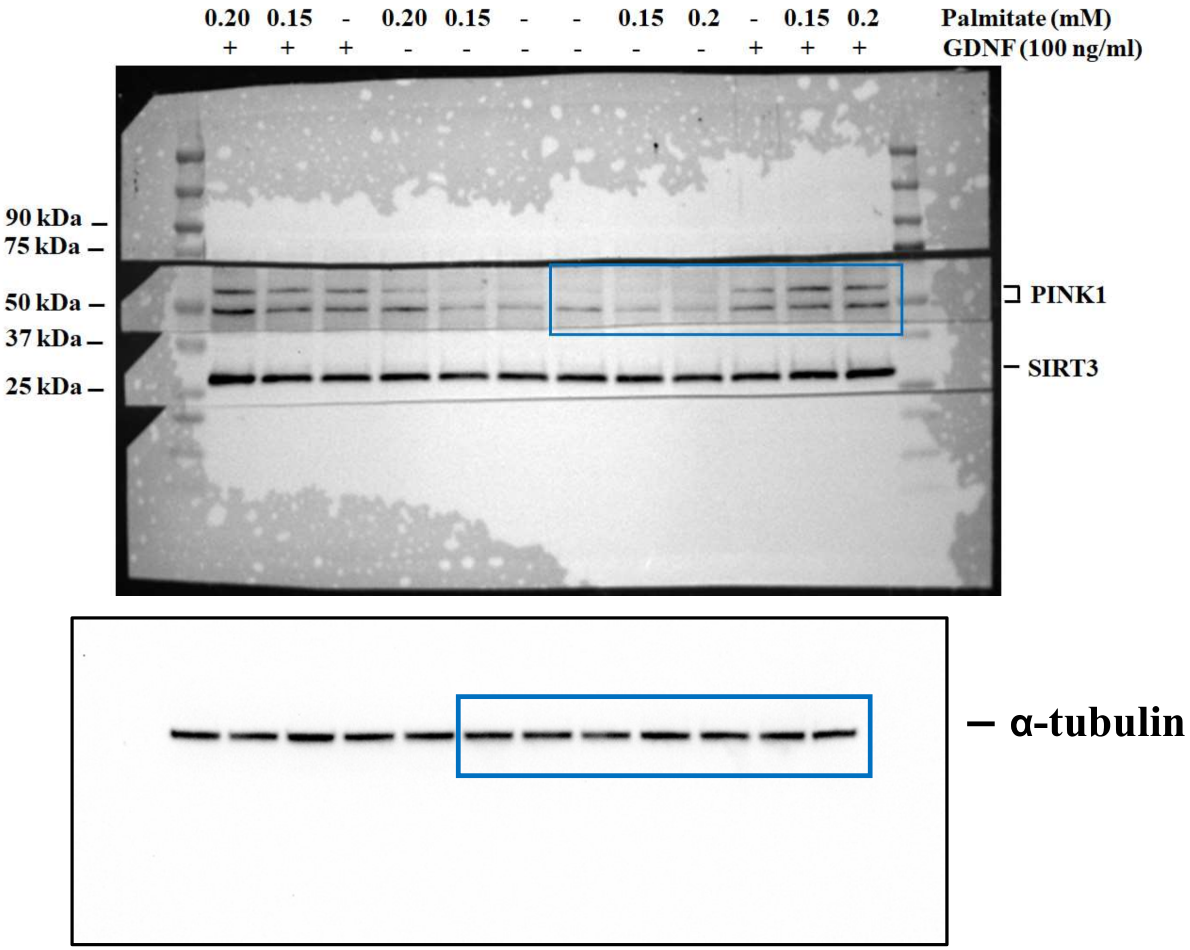

S4. Western blot analysis of PINK1 and α-tubulin (loading control) protein levels in primary human hepatocytes cultured for 24h in the presence and absence of palmitate (PA) and GDNF. Boxed area is where bands in Fig 3B were cropped from.

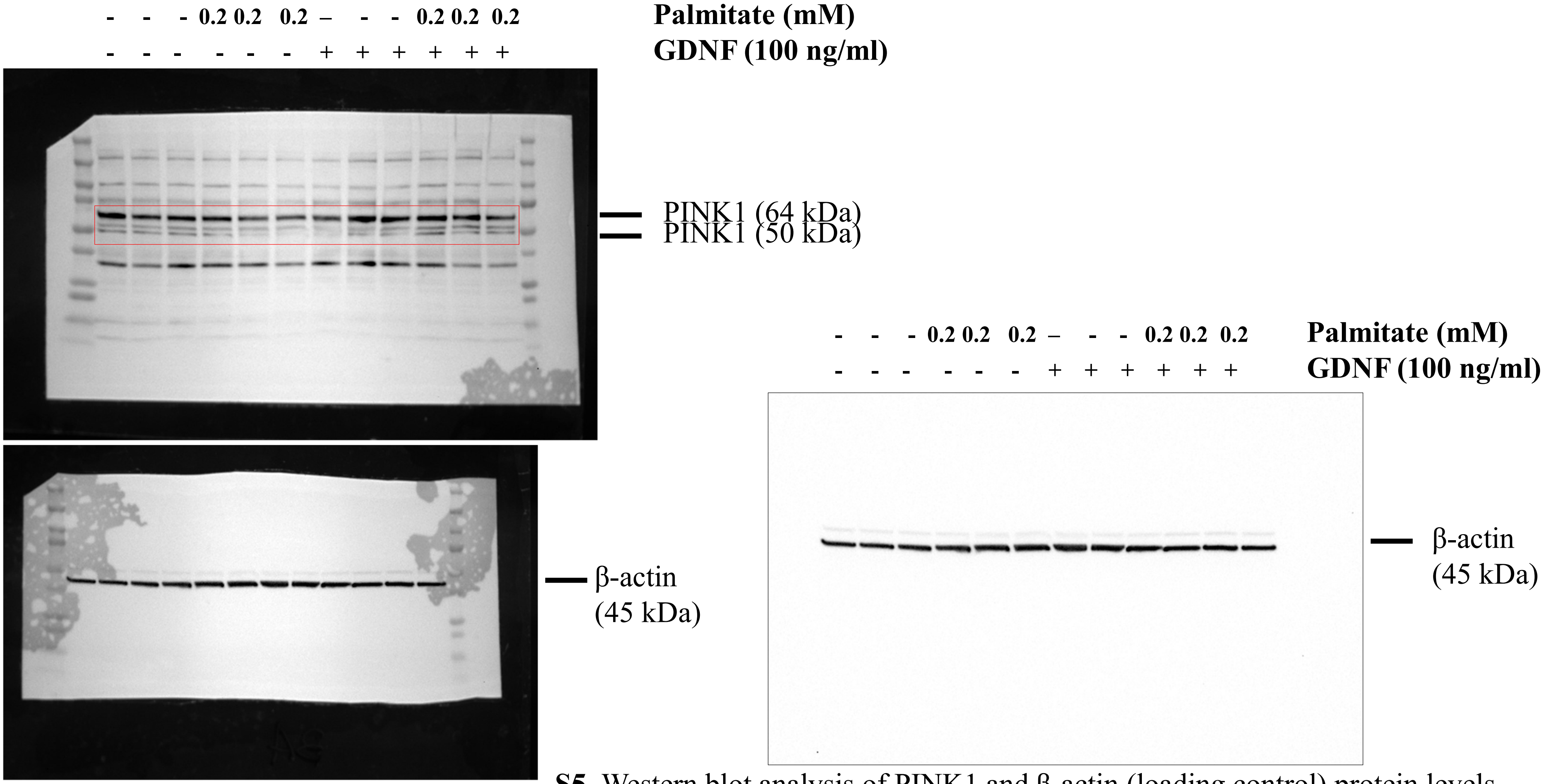

S5. Western blot analysis of PINK1 and β-actin (loading control) protein levels in rat hepatocytes exposed to palmitate (PA) and GDNF for 24h.

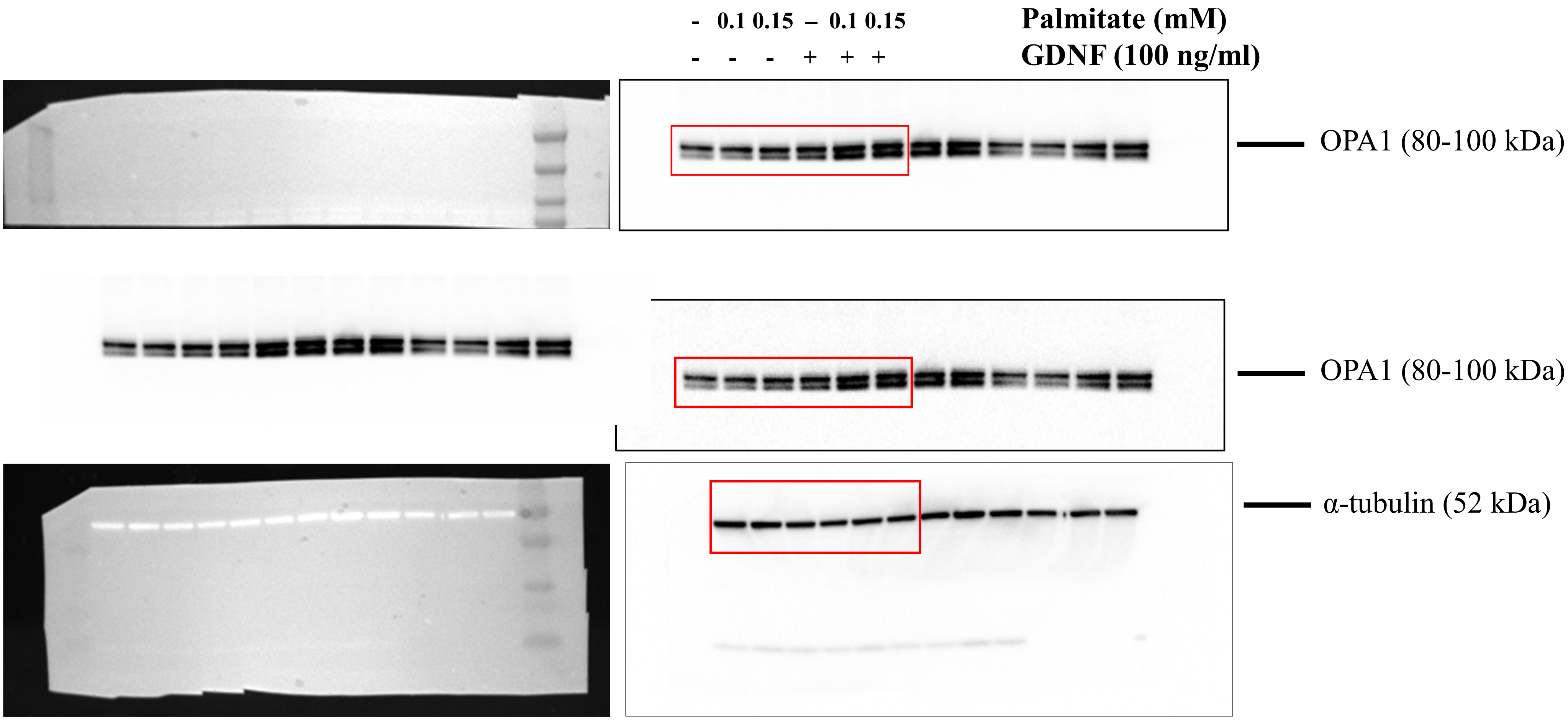

S6. Western blot analysis of OPA1 and α-tubulin (loading control) protein levels in rat hepatocytes exposed to Palmitate (PA) and GDNF for 24h.

ORIGINAL WESTERN BLOTS

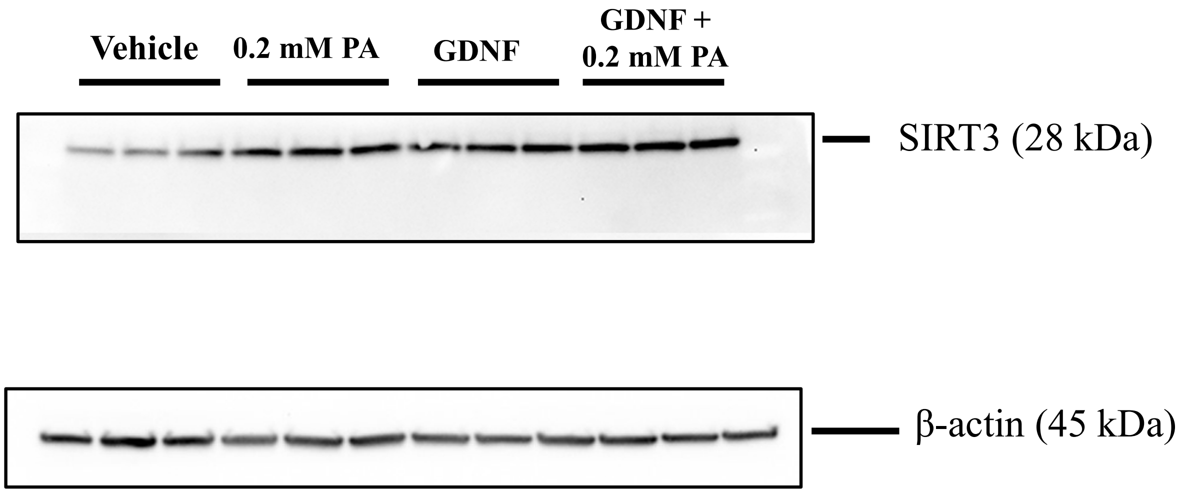

S7. Western blot analysis of SIRT3 and β-actin (loading control) protein levels in rat hepatocytes cultured for 24h in the presence and absence of palmitate (PA) and GDNF.

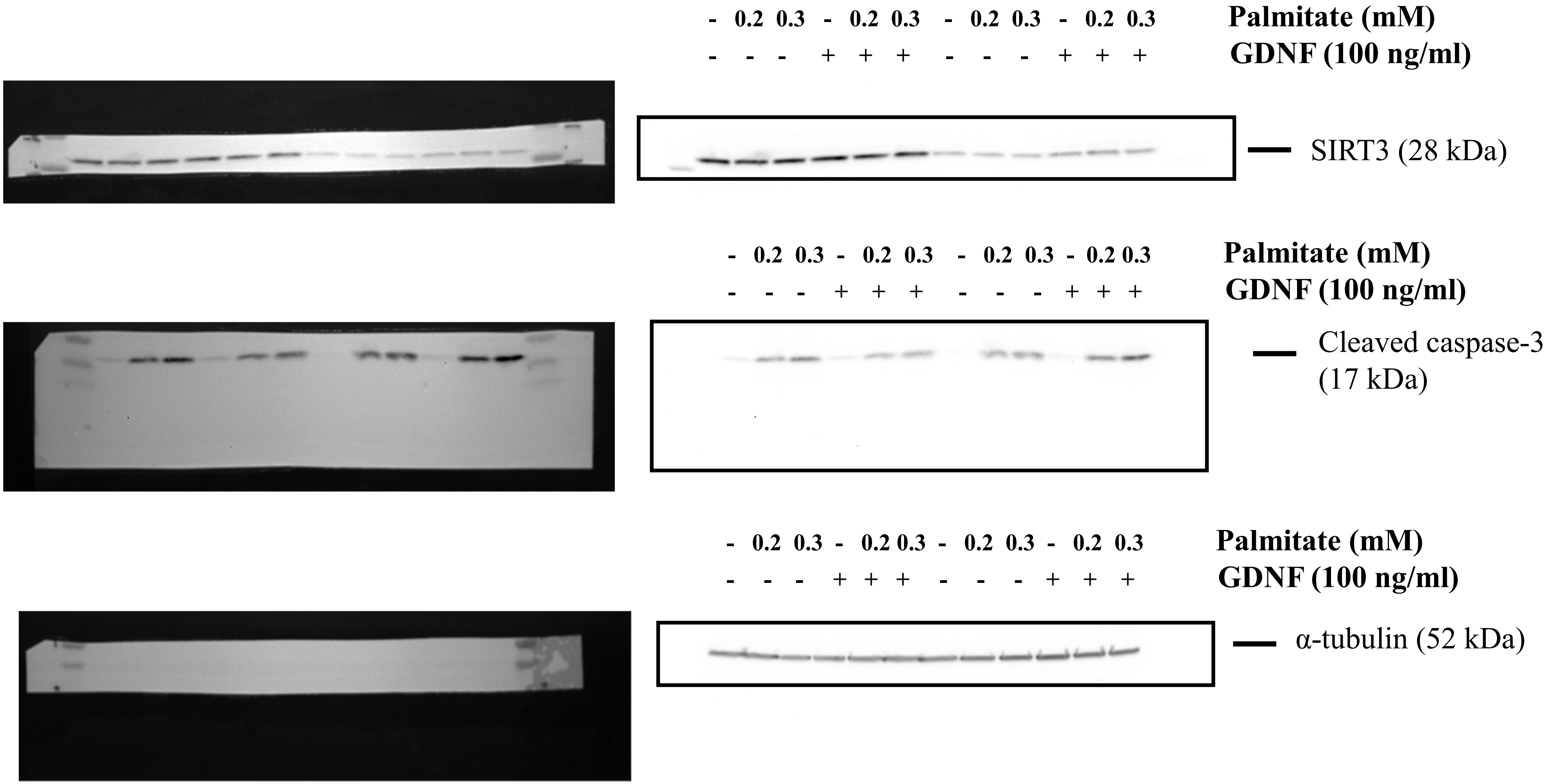

S8. Western blot analyses of SIRT3, cleaved caspase-3 and α-tubulin (loading control) levels in control and SIRT3 siRNA-transfected rat hepatocytes after 24h exposure to palmitate (PA) and GDNF.
